# Lean Body Mass, Muscle Architecture, and Performance in Well-Trained Female Weightlifters

**DOI:** 10.3390/sports8050067

**Published:** 2020-05-18

**Authors:** Nikolaos Zaras, Angeliki-Nikoletta Stasinaki, Polyxeni Spiliopoulou, Marios Hadjicharalambous, Gerasimos Terzis

**Affiliations:** 1 Human Performance Laboratory, Department of Life and Health Sciences, University of Nicosia, 46 Makedonitissas Ave., P.O. Box 24005, 1700 Nicosia, Cyprus; hadjicharalambous.m@unic.ac.cy; 2 Sports Performance Laboratory, School of Physical Education and Sport Science, National and Kapodistrian University of Athens, 41 Ethnikis Antistassis str., 172 37 Daphne, Athens, Greece; agstasin@phed.uoa.gr (A.-N.S.); spipolyxeni@phed.uoa.gr (P.S.); gterzis@phed.uoa.gr (G.T.)

**Keywords:** snatch, clean and jerk, muscle strength, muscle power, muscle hypertrophy, ultrasonography

## Abstract

Lean mass and quadriceps muscle architecture have been associated with performance in male well-trained weightlifters, but no data exist for female weightlifters. The aim of the study is to investigate the relationship between lean mass, quadriceps cross sectional area (CSA), and muscle architecture with weightlifting performance in female weightlifters. Eight well-trained female weightlifters (age 23.5 ± 6.3 years, maximum total lifting performance = 147.4 ± 34.1 kg) participated in the study. Five of the athletes were members of the national team and three were among the nation’s top-five performers of the respective body-weight category. Measurements included maximum lifting performance in snatch and clean and jerk, body composition (dual x-ray absorptiometry), vastus lateralis (VL) muscle architecture, vastus intermedius (VI) muscle thickness and quadriceps muscles’ CSA and countermovement jump (CMJ). Very large to nearly perfect correlations were found between snatch and clean and jerk for trunk lean body mass (r = 0.959 and 0.929), for total CSA (r = 0.732 and 0.608), and CMJ power (r = 0.933 and 0.896). These results suggest that lean body mass, quadriceps’ CSA and CMJ should be monitored regularly in female weightlifters to detect potential modifications in lifting performance.

## 1. Introduction

Olympic weightlifting includes two explosive movements: the snatch and the clean and jerk [1]. Both the snatch and the clean and jerk are multijoint movements where almost all the musculature system of the athlete must be activated for achieving a successful lift [2,3]. During these movements, weightlifters achieve the highest power output reported in sports [4]. Muscular power is largely determined by the fiber type composition of the contracting muscles, the neuromuscular facilitation level, and the lean muscle mass involved in a specific movement pattern [5]. Thus, athletes devote a large part of their training aiming to improve these parameters, although little is known about the association of these parameters and performance in well-trained weightlifters. Recently, a study with elite male weightlifters showed that lean body mass was correlated with performance in snatch (r = 0.727), clean and jerk (r = 0.791), and in total lifting capacity (r = 0.799) [6]. Similar results were presented in elite young male weightlifters where the lean body mass was estimated with skinfolds [7]. In contrast, Horsby et al. [8], did not observe any association between lean body mass and weightlifting performance in male and female athletes, while they reported minor changes in lean body mass after 20 weeks of training. Lean mass is distributed differently in the upper and lower body of males and females: the difference between genders tends to be larger in the upper extremities’ lean body mass, suggesting a much lower power capacity for the upper extremities in females compared to the lower extremities [9], although this issue has not been examined in weightlifters. Considering this, it is reasonable to hypothesize that lower body lean mass of female weightlifters would be of greater importance for performance than the upper body lean mass, further suggesting that special care should be taken to improve upper body musculature in these athletes. Therefore, it would be of practical interest to examine whether the upper and lower extremities’ lean body mass correlates with weightlifting performance in female well-trained athletes, in order to provide specific training directions for athletes and coaches. Nevertheless, the possible link between body part lean mass and performance in female weightlifters remains unknown.

Muscle architectural characteristics, namely muscle thickness, fascicle angle, and fascicle length, have been associated with athletic power performance [10,11,12]. However, very little is known about the impact of these parameters on weightlifting performance. Recently, significant correlations were reported between vastus lateralis (VL) muscle thickness and fascicle angle with total weightlifting performance after sixteen weeks of training in elite male weightlifters (Pearson’s r = 0.516 and 0.771, for thickness and angle, respectively) [6]. In contrast, a recent study in collegiate weightlifters failed to observe any correlations between muscle architecture and weightlifting performance following 20 weeks of training [8]. No other data exist regarding the relationship between muscle architecture and performance in weightlifters particularly in female athletes. 

The purpose of the present study was to investigate the relationship between lean body mass, muscle architecture, and weightlifting performance in well-trained female weightlifters. The hypothesis was that lean body mass and quadriceps’ muscle architectural characteristics would largely correlate with weightlifting performance in female athletes.

## 2. Materials and Methods

### 2.1. Athletes 

Eight well-trained female weightlifters (age 23.5 ± 6.3 years, age ranged 15–32 years, mass 63.3 ± 6.9 kg, height 1.64 ± 0.05 m, best performance in snatch 65.9 ± 15.4 kg, best performance in clean and jerk 81.5 ± 18.8 kg, best total lifting performance 147.4 ± 34.1 kg) volunteered to participate in the study. All athletes competed in national and international weightlifting competitions. Five athletes were members of the national team while the other three were classified in as the top-five athletes of the respective body-weight category during the previous national championship. Athletes self-reported not using illegal substances and were in good general health. They were informed about the risks and benefits of the study prior to entry and then signed an institutional approved informed consent document. Signed parental consent was also obtained for two athletes who were under 18 years of age. All procedures were in accordance with the 1975 Declaration of Helsinki as revised in 2000 and were approved by the institutional ethics committee (project number 1021/5/10/2017).

### 2.2. Muscle Architecture and Quadriceps’ Ultrasonography

Athletes reported to the laboratory during the morning hours, 48 h after the last training session. They remained in supine position for 15 min before ultrasonography evaluation. Ultrasound images for VL muscle architecture and vastus intermedius (VI) muscle thickness were obtained at the 50% of the distance from the central palpable point of the greater trochanter to the lateral condyle of the femur [13] of the dominant leg. B-mode axial-plane ultrasound images (Product model Z5, Shenzhen Mindray Bio-Medical Electronics Co., Ltd., Shenzhen, China) were taken with a 10 MHz linear-array probe (38-mm width) with an extended field of view mode [11,14] for capturing VL muscle thickness, fascicle angle, fascicle length as well as VI thickness. In each athlete, two images were assessed with an image analysis software (Motic Images Plus, 2.0, Hong Kong, China) and the mean value was used for statistical analysis.

Quadriceps’ cross sectional area (CSA) measurement was performed at 40% of the distance between the center of the patella and the medial aspect of the anterior superior iliac spine (proximal to the knee). A perpendicular guideline was drawn with an indelible marker, so that the probe was moved transversely across the thigh. Using the extended field of view mode, every image pictured the CSA of each of the four heads separately [15,16]. The CSA of each head (vastus lateralis, VL; rectus femoris, RF; vastus intermedius, VI; vastus medialis, VM) was assessed with image analysis software (Motic Images Plus, 2.0, Hong Kong, China). The CSA of whole quadriceps femoris was the sum of the four heads of the muscle group and presented here as total CSA (T). Two images were taken from each participant and the mean value was used for statistical analysis. 

### 2.3. Countermovement Jumping

Following the evaluation of muscle architecture, athletes performed the countermovement jumps (CMJs). After five minutes warm-up on the treadmill with 8 km/h and 4 submaximal CMJs with approximately 80%–90% of maximum intensity, athletes performed 4 maximal CMJs with 2 min rest between each attempt on a force platform (Applied Measurements Ltd. Co., Reading, UK; WP800, A/D sampling frequency 1 kHz) with arms akimbo. Data from the force platform were recorded and analyzed (Kyowa sensor interface PCD-320 A) for calculating maximum vertical jump height, power output, and velocity during the push off phase [17]. The jump with the best jumping height was used for analysis. 

### 2.4. Dual Energy X-Ray Absorptiometry

On the following day, after 12 h of fasting and before the morning training session, body composition was assessed via dual x-ray absorptiometry (DXA, model DPX-L; LUNAR Radiation, Madison, WI, USA). Athletes were placed supine on the DXA platform with minimal clothing. From the scan analysis total lean mass, legs lean mass, arms lean mass, and trunk lean mass were evaluated. All measurements were analyzed using the LUNAR radiation body composition program by an experienced researcher. 

### 2.5. Olympic Weightlifting Performance

Performance in weightlifting was measured at the training facilities of the national team during the afternoon hours at a standard temperature of ~24 °C [18]. Athletes performed a maximum one repetition maximum (1-RM) in snatch and clean and jerk according to the international regulations of the World Weightlifting Federation. Briefly, after a self-selected warm-up, athletes increased the resistance loads on the barbell until they failed to lift the external load. Three maximum attempts were given to athletes after 95% of the predicted 1RM in order to achieve their maximum. At all times, a certified coach was present for providing feedback to athletes. The best performance in all lifts was used for the statistical analysis. 

### 2.6. Statistics 

All data are presented as means ±SD. Pearson’s r product moment correlation coefficient was used to explore the relationships between weightlifting performance, CMJ, muscle architecture, and body composition. In addition, magnitude of effect for the correlations was based on the following scale: trivial <0.10; small <0.10–0.29; moderate ≤0.30–0.49; large ≤0.50–0.69; very large ≤0.70–0.89; and nearly perfect ≥0.9 [19]. Performance in snatch, clean and jerk, and total were transformed according to the Sinclair formula which is a polynomial equation for weightlifters and used as a method of obviating body mass differences in weightlifting totals [8,20]. Reliability for all measurements was performed using the intraclass correlation coefficients (ICCs) with 95% confidence interval (CI). Significance was accepted at *p* ≤ 0.05. All statistical analyses were performed using SPSS version 21.0 software (SPSS, Inc., Chicago, IL, USA).

## 3. Results

Reliability for weightlifting performance in snatch, clean and jerk, and total in female athletes was previously determined with coefficient of variation (CV) to be 3.7%, 3.5%, and 3.2%, respectively [21]. Additionally, Table 1 presents the ICC and CI for all measurements. 

All athletes completed the measurements without any injury. 1-RM strength in snatch, clean and jerk, and total as well as measurements of CMJ and lean mass are presented in Table 2. 

VL muscle thickness, angle, and fascicle length were 2.4 ± 0.4 cm, 17.3 ± 2.5°, and 8.8 ± 1.1 cm, respectively while VI thickness was 2.1 ± 0.5 cm CSA of VL, VI, VM, RF, and T were 23.2 ± 3.2 cm^2^, 29.1 ± 5.2 cm^2^, 8.7 ± 2.1 cm^2^, 9.1 ± 3.3 cm^2^, and 70.1 ± 11.6 cm^2^, respectively. 

**Figure 1:**
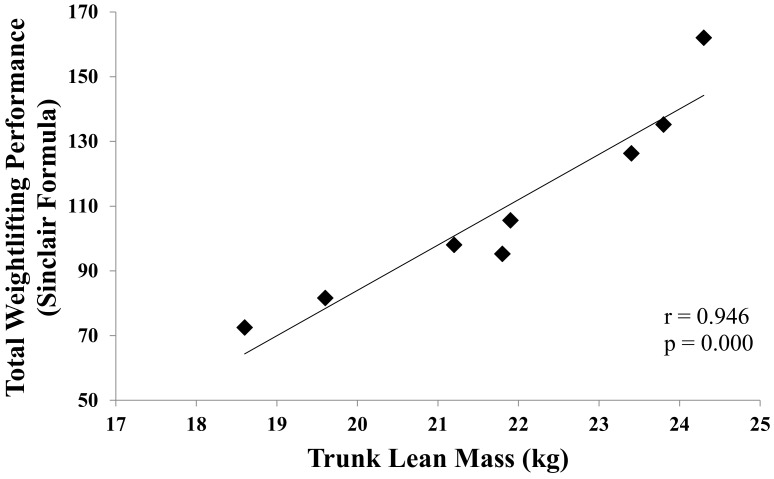
Correlation between total weightlifting performance expressed with Sinclair formula and trunk lean mass in 8 competitive female weightlifters.

Very large to nearly perfect positive correlations were found between lean mass and weightlifting performance (Table 3). Higher correlations were observed between weightlifting performance and trunk lean mass (Figure 1). Additionally, very large positive correlations were found between weightlifting performance and CMJ height, power, power per body mass, and velocity (Table 3). Correlations between weightlifting performance and VL muscle architecture, VI muscle thickness as well as with quadriceps’ CSA are presented in Table 4. 

CMJ power was significantly correlated with total lean mass (r = 0.851), legs lean mass (r = 0.900), arms lean mass (r = 0.708), and trunk lean mass (r = 0.906). Furthermore, positive correlations were observed between CMJ power and VL thickness (r = 0.540), fascicle angle (r = 0.470), fascicle length (r = 0.658), and VI thickness (r = 0.428). Finally, positive correlations were observed between CMJ power and VL CSA (r = 0.618), VI CSA (r = 0.797), VM CSA (r = 0.506), and RF (r = 0.351).

## 4. Discussion

The purpose of the study was to investigate the relationship between weightlifting performance, lean mass, and quadriceps’ muscle architecture in well-trained female weightlifters. The main finding was that lean body mass was closely correlated with weightlifting performance in these athletes, suggesting that regular evaluation of lean mass might be a valuable tool to monitor training-induced adaptations relative to weightlifting performance. In line with this result, previous studies in both young and senior elite male weightlifters showed significant positive correlations between weightlifting performance and lean body mass [6,7]. Trunk musculature includes the back extensors which are well developed in weightlifters [22]; however, this is the first time that the lean mass of this body part is related to weightlifting performance in female athletes. Moreover, the lower body lean mass presented slightly lower correlation coefficients with weightlifting performance compared to the upper body lean mass. Recent data presented slightly larger correlation coefficients between upper body lean mass and total weightlifting performance compared to the lower body lean mass in male weightlifting athletes [6]. These contrasting but consistent results may suggest that female weightlifters could rely more on the lower body musculature for performance compared to male athletes because of their presumably greater deficit in the upper body musculature/power compared to their male counterparts [9], which may be further reinforced by periodized resistance training [23]. However, such a premise must be further examined. 

Total quadriceps CSA, as revealed with ultrasonography, presented higher correlation with weightlifting performance compared to the CSA of each individual quadriceps’ head. Yet, this correlation was not so precise as the one provided by the lean mass of the lower extremities and especially the trunk lean mass. This suggests that ultrasonographic measurement of the CSA may not be of greater value for weightlifting performance compared to the DXA lean mass evaluation. Both the snatch and the clean and jerk require the recruitment and power production of the neuromuscular system of almost all body parts [2,3]. Therefore, the results of the present study suggest that quadriceps’ CSA per se may not provide a valid prediction of weightlifting performance in female weightlifters. Interestingly, VI CSA was more highly correlated with weightlifting performance compared to VL, VM, and RF. However, more data are needed to draw confident conclusions about the contribution of the quadriceps’ muscle heads in weightlifting performance. 

The moderate correlation coefficients found between VL architecture and weightlifting performance suggests that VL thickness, fascicle angle, and fascicle length may not provide additional important information regarding the training condition of a female weightlifter. Similar results were obtained in a recent study showing that VL thickness, angle, and fascicle length were not strongly related to weightlifting performance in male athletes before and after 16 weeks of training [6]. Correlation coefficients of similar strength were also reported between VL muscle thickness, medial gastrocnemius muscle thickness and angle, and power clean performance [24]. This reinforces that the quadriceps muscle alone may not provide a valid estimate of weightlifting ability either in male or female athletes. 

As expected, CMJ performance was significantly correlated with snatch, clean and jerk and total lifting capacity in female athletes. This test is a valid index for evaluating lower body power performance [25,26]. In agreement with the present findings, power production during the CMJ was correlated with weightlifting performance in both male and female athletes of different age, gender, and performance level [27]. The current results re-emphasize the use of the CMJ for regular monitoring of the lower body power capacity and weightlifting performance in both male and female athletes. In addition, total lean mass was very closely correlated with CMJ power production while quadriceps’ CSA and especially VI CSA were closely correlated with CMJ power. These results underpin the role of lean mass in power production in female weightlifters. 

The current study describes the relationship between lean mass, muscle architecture, quadriceps’ CSA, and weightlifting performance in female weightlifters of national and international level. Olympic weightlifting is an explosive sporting event where muscle fiber types and neural factors may contribute to performance. However, in the current study neither fiber type composition nor electromyographic activity was examined, which might have provided a better understanding of the results. Another limitation of the study was that the state of hydration and nutrition of the athletes was only monitored before DXA evaluation. Further studies should examine the impact of the architectural characteristics of other protagonist muscles of the upper or trunk muscles in larger groups of elite athletes, providing lower confidence intervals in order to gain a better understanding of weightlifting’s reliance on these neuromuscular factors. 

## 5. Conclusions

In conclusion, lean body mass is closely correlated with weightlifting performance in well-trained female athletes. The lean body mass of the trunk seems to be the best predictor of weightlifting performance. VL muscle architecture may not provide additional information for performance and the same holds true for the quadriceps’ T CSA compared to lean mass. The CMJ height, power, and velocity may provide good estimates of weightlifting performance in both female and male athletes. Consequently, coaches and athletes may regularly monitor modifications in lean body mass and CMJ for predicting potential changes in weightlifting performance in female athletes. Additionally, periodized training programs may focus on enhancing lower body lean mass and maximum strength in order to enhance weightlifting performance. As a final point, changes in CMJ following a training mesocycle may suggest increases in lean mass, quadriceps muscle hypertrophy, and weightlifting performance. Thus, when DXA or muscle ultrasound access is difficult, CMJ may provide useful insights into the rate of training adaptations. 

## Figures and Tables

**Table 1:** Intra class correlation coefficient and confidence intervals for the variables included in the study.

Variables	ICC	Lower Bound	Upper bound	Sig.
CMJ_height_	0.970	0.850	0.993	0.001
CMJ_power_	0.990	0.991	0.998	0.000
CMJ_w/kg_	0.989	0.987	0.997	0.000
CMJ_velocity_	0.967	0.850	0.993	0.001
Total lean mass	0.995	0.966	0.999	0.000
Legs lean mass	0.997	0.986	0.999	0.000
Trunk lean mass	0.969	0.789	0.992	0.001
Arms lean mass	0.990	0.931	0.999	0.000
VL thickness	0.970	0.856	0.987	0.001
VL angle	0.880	0.609	0.965	0.001
VL fascicle length	0.840	0.470	0.955	0.001
VI thickness	0.928	0.799	0.975	0.001
VL CSA	0.962	0.835	0.991	0.001
VI CSA	0.956	0.814	0.99	0.000
VM CSA	0.872	0.479	0.971	0.001
RF CSA	0.949	0.725	0.989	0.001
T CSA	0,974	0.892	0.994	0.000

ICC = intra class correlation coefficient; CMJ = countermovement jump; VL = vastus lateralis; VI = vastus intermedius; VM = vastus medialis; RF = rectus femoris; T = total; CSA = cross sectional area; Sig. = significance.

**Table 2:** Results from weightlifting, CMJ, and DXA measurements of the 8 female weightlifters.

Performance and Body Composition	Values
Snatch (kg)	63.8 ± 16.2
Clean and jerk (kg)	79.4 ± 18.7
Total (kg)	143.2 ± 34.7
CMJ_height_ (cm)	29.6 ± 5.3
CMJ_power_ (W)	2623.1 ± 418.7
CMJ_w/kg_ (W/kg)	41.4 ± 4.2
CMJ_velocity_ (m/sec)	2.5 ± 0.4
Total lean mass (kg)	45.9 ± 3.9
Legs lean mass (kg)	16.5 ± 1.6
Trunk lean mas (kg)	21.8 ± 2.0
Arms lean mass (kg)	5.14 ± 0.5

CMJ = countermovement jump; DXA = dual energy X-ray absorptiometry.

**Table 3:** Correlation coefficients (Pearson’s r) between weightlifting performance and lean mass and CMJ in eight well-trained female weightlifters.

Weightlifting	Total Lean Mass	Legs Lean Mass	Arms Lean Mass	Trunk Lean Mass	CMJ Height	CMJ Power	CMJ P·kg^−1^	CMJ Velocity
Snatch	0.853 ^#^	0.872 ^#^	0.835 ^#^	0.959 *	0.756 ^#^	0.933 *	0.879 ^#^	0.751 ^#^
Clean and Jerk	0.791 ^#^	0.809 ^#^	0.802 ^#^	0.929 *	0.816 ^#^	0.896 ^#^	0.919 *	0.811 ^#^
Total	0.823 ^#^	0.841 ^#^	0.820 ^#^	0.946 *	0.791 ^#^	0.916 *	0.903 *	0.785 ^#^

CMJ = countermovement jump. ⁺ trivial <0.10; ˠ small <0.10–0.29; ^†^ moderate ≤0.30–0.49; ^‡^ large ≤0.50–0.69; ^#^ very large ≤0.70–0.89; * nearly perfect ≥0.9; P·kg^−1^ = CMJ power per kg of body weight.

**Table 4:** Correlation coefficients (Pearson’s r) between weightlifting performance and VL muscle architecture, VI thickness, and quadriceps’ CSA, in eight female well-trained weightlifters.

Weightlifting	VL Thickness	VL Angle	VL Length	VI Thickness	CSA VL	CSA VI	CSA VM	CSA RF	CSA Total
Snatch	0.430 ^†^	0.459 ^†^	0.517 ^‡^	0.151 ^ˠ^	0.361 ^†^	0.624 ^‡^	0.241 ^ˠ^	0.610 ^‡^	0.732 ^#^
Clean and Jerk	0.337 ^†^	0.436 ^†^	0.414 ^†^	0.094 ⁺	0.271 ^ˠ^	0.593 ^‡^	0.137 ^ˠ^	0.565 ^‡^	0.680 ^‡^
Total	0.381 ^†^	0.448 ^†^	0.464 ^†^	0.121 ^ˠ^	0.314 ^†^	0.609 ^‡^	0.186 ^ˠ^	0.588 ^‡^	0.706 ^#^

CSA = cross sectional area; VL = vastus lateralis; VI = vastus intermedius; VM = vastus medialis; RF = rectus femoris. ⁺ trivial <0.10; ^ˠ^ small <0.10–0.29; ^†^ moderate ≤0.30–0.49; ^‡^ large ≤0.50–0.69; ^#^ very large ≤0.70–0.89; * nearly perfect ≥0.9.
